# Long-term vitamin K antagonists treatment patterns of Non-Valvular Atrial Fibrillation (NVAF): a population-based cohort study

**DOI:** 10.1186/s12872-016-0269-4

**Published:** 2016-05-10

**Authors:** Christel Renoux, Janie Coulombe, Samy Suissa

**Affiliations:** Centre for Clinical Epidemiology, Lady Davis Institute for Medical Research, Jewish General Hospital, 3755 Cote Ste-Catherine, H-461, Montréal, Québec H3T 1E2 Canada; Department of Neurology and Neurosurgery, McGill University, Montréal, Québec Canada; Department of Epidemiology and Biostatistics, McGill University, Montréal, Québec Canada

**Keywords:** Atrial fibrillation, Anticoagulants, Sex, Cohort studies, Epidemiology

## Abstract

**Background:**

Recent trends in vitamin K antagonists (VKA) use in non-valvular atrial fibrillation (NVAF) are useful to evaluate the potential improvement in management of NVAF since the introduction of new oral anticoagulants. Our objective was therefore to describe the contemporary VKA treatment patterns following NVAF diagnosis.

**Methods and Results:**

We used the computerized databases of the Régie de l’assurance maladie du Québec (RAMQ), responsible for administering the universal health care services for all its residents, to identify a population-based cohort of 135,241 patients with an incident diagnosis of NVAF during 2000–2009 and RAMQ medication coverage.

Following NVAF diagnosis, 47.1 % of the patients were prescribed VKA, 35.5 % received an antiplatelet only, and 17.4 % did not initiate antithrombotic therapy. The proportion of patients initiating VKA within 3 months of diagnosis increased from 33 % to 39 % over the 10-year study period, mainly driven by a higher proportion of treated patients aged 80 or more (from 29 % to 41 %). At the end of the study period, women were prescribed VKA as frequently as men, except in the subgroup of patients with a low risk of ischemic stroke. The median time from VKA initiation to the first discontinuation varied greatly according to the definition of discontinuation, ranging from 11 months to 5.7 years.

**Conclusion:**

Although VKA remain underused after NVAF diagnosis, there has been an increase in VKA treatment over the last decade, particularly among older patients. Also the gap in treatment between men and women has been closing within the last decade. Once initiated, most VKA interruptions were temporary rather than definitive.

## Background

Anticoagulants are prescribed to prevent ischemic stroke in patients with non-valvular atrial fibrillation (NVAF) [1]. These include vitamin K antagonists (VKA) such as warfarin that have several side-effects including bleeding, drug-drug interactions, and a narrow therapeutic window that necessitates monitoring to keep patients within the recommended INR range. Therefore, despite proven effectiveness of anticoagulation in the prevention of thromboembolic events, many patients with NVAF remain untreated. A recent systematic review showed that VKA are also underused in the subgroup of patients with a high risk of ischemic stroke [2].

The recent introduction of new oral anticoagulants in the market may improve the overall management of patients diagnosed with NVAF. Indeed, based on results of randomised controlled trials, these new molecules are as effective as VKA in preventing ischemic stroke while producing less intracerebral haemorrhage and being more convenient to use [3]. In order to evaluate their impact both in terms of the proportion of patients initiating anticoagulation after NVAF diagnosis and long-term compliance to treatment, updated estimates of anticoagulation patterns with VKA are necessary for meaningful comparison. However, few studies have assessed long-term anticoagulation management with VKA and have examined recent trends of VKA use over time.

The objectives of this study were therefore to describe the VKA treatment patterns following NVAF diagnosis in a population-based cohort with long-term follow-up, identified using the Canadian Province of Québec’s health insurance databases.

## Methods

### Source of data

This study was conducted using the computerized databases of the *Régie de l'assurance maladie du Québec* (RAMQ), the *Maintenance et exploitation des données pour l’étude de la clientèle hospitalière* (MED-ÉCHO), and the *Institut de la statistique du Québec* (ISQ). Health care coverage is mandatory for all Québec residents except visitors, non-Canadian students, and individuals residing outside of Québec for more than 183 days in the year who are not eligible for coverage. The RAMQ, which is responsible for administering these universal health care services, maintains three computerised databases. The *demographic database* contains the age, sex and postal code of all individuals registered. The *medical services database* contains information on the medical services, including nature of the service rendered, specialty of treating and referring physician, date and location, as well as the diagnostic code of the service (International Classification of Diseases, 9th Revision, Clinical Modification (ICD-9-CM) or enhanced version of ICD-10 for Canada ICD-10-CA). This program is universal for all Québec residents and is fee-for-service. The *prescription database* contains information on out-patient prescription medications including name, dose and amount of drug dispensed, date, prescribed number of days of treatment, and whether it was a refill or a new prescription. This fee-for-service program (the pharmacy claims reimbursement for the drugs dispensed) covers all individuals 65 years of age and older, welfare recipients, and since 1996, extends to all Québec residents who do not have private medication insurance or who choose to be covered by the RAMQ program. MED-ÉCHO maintains the *hospital inpatient database* which contains data pertaining to all Québec hospitalisations (including day surgery and inpatient stays), such as date and type of admission and discharge, type of establishment, one primary and secondary diagnoses, as well as procedure codes (with corresponding dates). Prior to 2006, diagnoses were classified according to the ICD-9-CM and procedures were coded according to the Canadian Classification of Diagnostic, Therapeutic, and Surgical Procedures (CCDTC). Since 2006, diagnoses and procedures are coded according to ICD-10-CA and the Canadian Classification of Health Interventions (CCI), respectively. Finally, the *cause of death database,* administered by ISQ, contains the date and cause of death, as well as the establishment where the death took place. Each of these databases contains the individual's *numéro d'assurance-maladie* (health insurance number), a unique number acquired at birth or at the time of residency, used for record linkage within the RAMQ databases and with MED-ECHO. The general accuracy of linkage between the prescription and the medical services databases was found to be 98.2 %, unfeasible linkages arising primarily from name changes, and the quality of the data has been documented [4, 5].

### Cohort definition

From the source population of all individuals in the RAMQ database, we first identified all patients, at least 18 years of age, with an inpatient or outpatient diagnosis for atrial fibrillation (ICD-9: 427.3, 427.31, 427.32; ICD-10: I48, I48.0, I48.1) between January 1, 2000 and December 31, 2009. Cohort entry (time zero) for all patients was defined at the date of the first diagnosis of NVAF. If the diagnosis occurred during a hospitalisation, cohort entry was set as the date of hospital discharge. To confirm the incident nature of the NVAF diagnosis, all subjects with any mention of AF in the two years prior to cohort entry diagnosis were excluded. Patients with a history of valvular aortic or mitral heart disease, previous valvular repair, or hyperthyroidism (either a treatment or a diagnosis) in the two years prior to cohort entry were also excluded. All cohort members were required to have RAMQ medication coverage for at least two years prior to cohort entry in order to provide sufficient baseline information on comorbidities and prior medication use. In addition, we excluded all patients with a prescription for VKA in the year prior to cohort entry. All cohort members were followed until RAMQ deregistration, death or end of the study period (December 31, 2009), whichever occurred first. The occurrence of death was determined from RAMQ and ISQ. MED-ÉCHO was used to identify in-hospital deaths.

### Exposure definition

To assess treatment patterns, all outpatient prescriptions for oral anticoagulants and antiplatelet agents (such as aspirin and clopidogrel) dispensed during follow-up were identified. All oral anticoagulants prescriptions were vitamin K antagonists (VKA) and will therefore be referred to as VKA. For each prescription, the date of dispensing together with product name, formulation, dose and duration was obtained. The duration of exposure to each drug was computed by assessing the continuity of successive prescriptions, considered as continuous use if the end of a prescription was followed by the start of the next prescription of the drug. To account for delays in refilling and reduced patient compliance, a 100 % grace period was added to the end of each prescription (100 % of the duration of that prescription) in assessing continuity of use. Thus, the duration of continuous use was the time between the date of the start of the first prescription after the diagnosis of NVAF and the last dispensing for which gaps between two repeated prescriptions were less than 100 % of the first of the two. A 100 % grace period was chosen as the median duration of individual prescriptions was only 10 days for VKA. Sensitivity analyses with the grace period set to 30 and 60 days were also carried out in order to assess the impact of different grace periods on the estimated duration of use until first discontinuation. Since RAMQ does not collect data on inpatient prescriptions, this study only provides data on outpatient medication use patterns.

### Data analysis

The study cohort was characterised by age, sex, comorbidities, Charlson comorbidity index, CHADS_2_ and CHA_2_DS_2_-VASc risk scores [6, 7] at the time of cohort entry using descriptive statistics. Comorbidities were measured during the 2 years prior to cohort entry. Kaplan-Meier technique was used to estimate the median time from NVAF diagnosis to VKA initiation and the median duration of initial use until first treatment interruption, modified for competing risks to account for deaths occurring during follow-up [8], and censored at the date of major bleeding [8]. Cox proportional hazards models were used to estimate the hazard ratio (HR) of treatment delay and treatment duration as a function of baseline patient characteristics, including age, sex, and baseline comorbidities. CHADS_2_ and CHA_2_DS_2_-VASc risk scores were not included in multivariate models in order to assess the independent effect of risk factors composing these scores. Follow-up was censored at the date of death, major bleeding, or the end of the study period (or at 3 months when assessing treatment delay). To assess trends of VKA initiation following NVAF over the 10-year study period, a logistic regression model was used with adjustment for age and CHADS_2_ score.

Confidence intervals were calculated using a significance level of 5 %. All statistical procedures were performed using SAS version 9.2 (SAS Institute Inc., Cary, North Carolina).

## Results

**Fig. 1:**
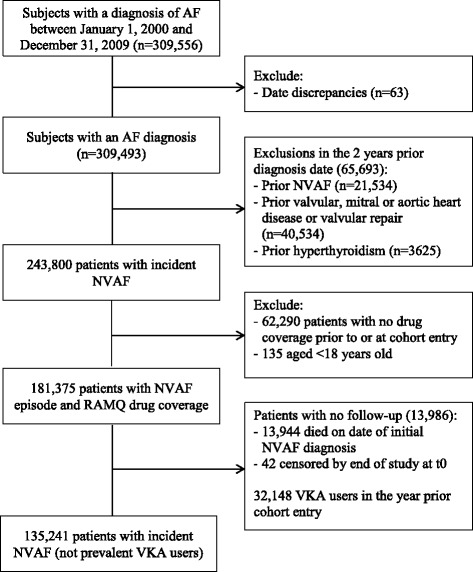
Details of incident non-valvular atrial fibrillation (NVAF) cohort definition

The source population included 309,556 subjects with at least one atrial fibrillation diagnosis code during the period 2000-2009. From these, we identified 135,241 patients with a first NVAF diagnosis, satisfying cohort inclusions and exclusions, and having drug insurance coverage for a minimum of two years at the time of cohort entry (Fig. 1). Table 1 describes the baseline characteristics of this incident NVAF cohort. NVAF was a hospital diagnosis in 47.8 % of the patients and the mean follow-up was 3.3 years. The mean age at NVAF diagnosis was 75.1 years, 74.8 % of the patients were 70 years or older at diagnosis, and 47.9 % were males. Women were older at NVAF diagnosis, and had a higher CHADS_2_ score at baseline. However, the prevalence of most comorbidities such as myocardial infarction, diabetes, hyperlipidemia, and chronic renal failure was higher in males compared to women. Patient initiating an antithrombotic treatment within 3 months following NVAF diagnosis were older and had more cardio- and cerebrovascular comorbidities than patients who were not prescribed VKA or antiplatelets.

**Table 1:** Baseline characteristics of the incident NVAF cohort

	Total	Men	Women	No initiation (within 3 months)	VKA* initiation	Antiplatelets* (only) initiation
	*N (%)*	*N (%)*	*N (%)*	*N (%)*	*N (%)*	*N (%)*
Cohort size	135,241	64,786 (47.90)	70,455 (52.10)	34,659 (25.63)	51,794 (38.30)	48,788 (36.07)
Age, mean (SD)	75.09 (11.41)	73.29 (11.30)	76.73 (11.26)	72.54 (14.65)	75.95 (8.85)	75.98 (10.92)
18-49	4,526 (3.35)	2,643 (4.08)	1,883 (2.67)	2,918 (8.42)	525 (1.01)	1,083 (2.22)
50-59	6,770 (5.01)	3,948 (6.09)	2,822 (4.01)	2,558 (7.38)	1,697 (3.28)	2,515 (5.15)
60-69	22,730 (16.81)	12,736 (19.66)	9,994 (14.18)	6,027 (17.39)	8,408 (16.23)	8,295 (17.00)
70-79	51,018 (37.72)	26,376 (40.71)	24,642 (34.98)	10,977 (31.67)	22,533 (43.51)	17,508 (35.89)
80-89	40,769 (30.15)	16,403 (25.32)	24,366 (34.58)	9,369 (27.03)	16,503 (31.86)	14,897 (30.53)
≥90	9,428 (6.97)	2,680 (4.14)	6,748 (9.58)	2,810 (8.11)	2,128 (4.11)	4,490 (9.20)
Comorbidities						
Hypertension	83,393 (61.66)	36,683 (56.62)	46,710 (66.30)	17,682 (51.02)	33,651 (64.97)	32,060 (65.71)
Myocardial infarction	27,909 (20.64)	16,186 (24.98)	11,723 (16.64)	3,475 (10.03)	10,081 (19.46)	14,353 (29.42)
Congestive heart failure	27,673 (20.46)	14,025 (21.65)	13,648 (19.37)	5,035 (14.53)	11,325 (21.87)	11,313 (23.19)
Prior stroke or TIA	11,654 (8.62)	5,644 (8.71)	6,010 (8.53)	1,726 (4.98)	5,088 (9.82)	4,840 (9.92)
Prior hemorrhage	21,080 (15.59)	11,160 (17.23)	9,920 (14.08)	6,452 (18.62)	6,437 (12.39)	8,191 (16.79)
Diabetes	34,337 (25.39)	17,893 (27.62)	16,444 (23.34)	6,318 (18.23)	13,688 (26.43)	14,331 (29.37)
Hyperlipidemia	57,433 (42.47)	30,360 (46.86)	27,073 (38.43)	8,165 (23.56)	23,688 (45.74)	25,580 (52.43)
Peripheral vascular disease	16,397 (12.12)	9,857 (15.21)	6,540 (9.28)	2,667 (7.69)	5,900 (11.39)	7,830 (16.05)
Chronic renal failure	14,467 (10.70)	8,228 (12.70)	6,239 (8.86)	3,124 (9.01)	4,960 (9.58)	6,383 (13.08)
Cancer	26,479 (19.58)	15,363 (23.71)	11,116 (15.78)	8,308 (23.97)	8,538 (16.48)	9,633 (19.74)
Charlson index, median (IQR)	1 (0-3)	1 (0-3)	1 (0-2)	1 (0-3)	1 (0-2)	1 (0-3)
0	50,090 (37.04)	21,300 (32.88)	28,790 (40.86)	14,295 (41.24)	20,308 (39.21)	15,487 (31.74)
1-2	49,213 (36.39)	23,036 (35.56)	26,177 (37.15)	11,517 (33.23)	19,606 (37.85)	18,090 (37.08)
≥3	35,938 (26.57)	20,450 (31.57)	15,488 (21.98)	8,847 (25.53)	11,880 (22.94)	15,211 (31.18)
CHADS_2_, median (IQR)	2 (1-3)	2 (1-2)	2 (1-3)	1 (1-2)	2 (1-3)	2 (1-3)
0	19,052 (14.09)	10,719 (16.55)	8,333 (11.83)	8,280 (23.89)	5,466 (10.55)	5,306 (10.88)
1	37,744 (27.91)	19,085 (29.46)	18,659 (26.48)	10,741 (30.99)	14,355 (27.72)	12,648 (25.92)
≥2	78,445 (58.00)	34,982 (54.00)	43,463 (61.69)	15,638 (45.12)	31,973 (61.73)	30,834 (63.20)
CHA_2_DS_2_-VASc, median (IQR)	3 (2-5)	3 (2-4)	4 (3-5)	3 (2-4)	4 (3-5)	4 (3-5)
0	4,209 (3.11)	4,209 (6.50)	0 (0.0)	2,212 (6.38)	875 (1.69)	1,122 (2.30)
1	11,530 (8.53)	7,962 (12.29)	3,568 (5.06)	4,973 (14.35)	3,380 (6.53)	3,177 (6.51)
≥2	119,502 (88.36)	52,615 (81.21)	66,887 (94.94)	27,474 (79.27)	47,539 (91.78)	44,489 (91.19)
HAS-BLED, median (IQR)	2 (1-3)	2 (1-3)	2 (1-3)	1 (1-2)	2 (1-3)	2 (2-3)
≤2	96,763 (71.55)	45,097 (69.61)	51,666 (73.33)	29,400 (84.83)	37,215 (71.85)	30,148 (61.79)
≥3	38,478 (28.45)	19,689 (30.39)	18,789 (26.67)	5,259 (15.17)	14,579 (28.15)	18,640 (38.21)
Medications						
Antihypertensive drugs**	99,313 (73.43)	45,477 (70.20)	53.836 (76.41)	19,384 (55.93)	40,750 (78.68)	39,179 (80.30)
NSAIDs	49,164 (36.35)	21,617 (33.37)	27,547 (39.10)	12,496 (36.05)	19,003 (36.69)	17,665 (36.21)
Statins	52,132 (38.55)	27,934 (43.12)	24,198 (34.35)	6,873 (19.83)	21,703 (41.90)	23,556 (48.28)

*Initiation within 3 months following NVAF diagnosis

**Refers to angiotensin-converting enzyme inhibitors, angiotensin receptor blockers, calcium channel blockers and diuretics

*Abbreviations*: *TIA* transient ischemic attack, *NSAIDs* non-steroidal anti-inflammatory drugs

**Fig. 2:**
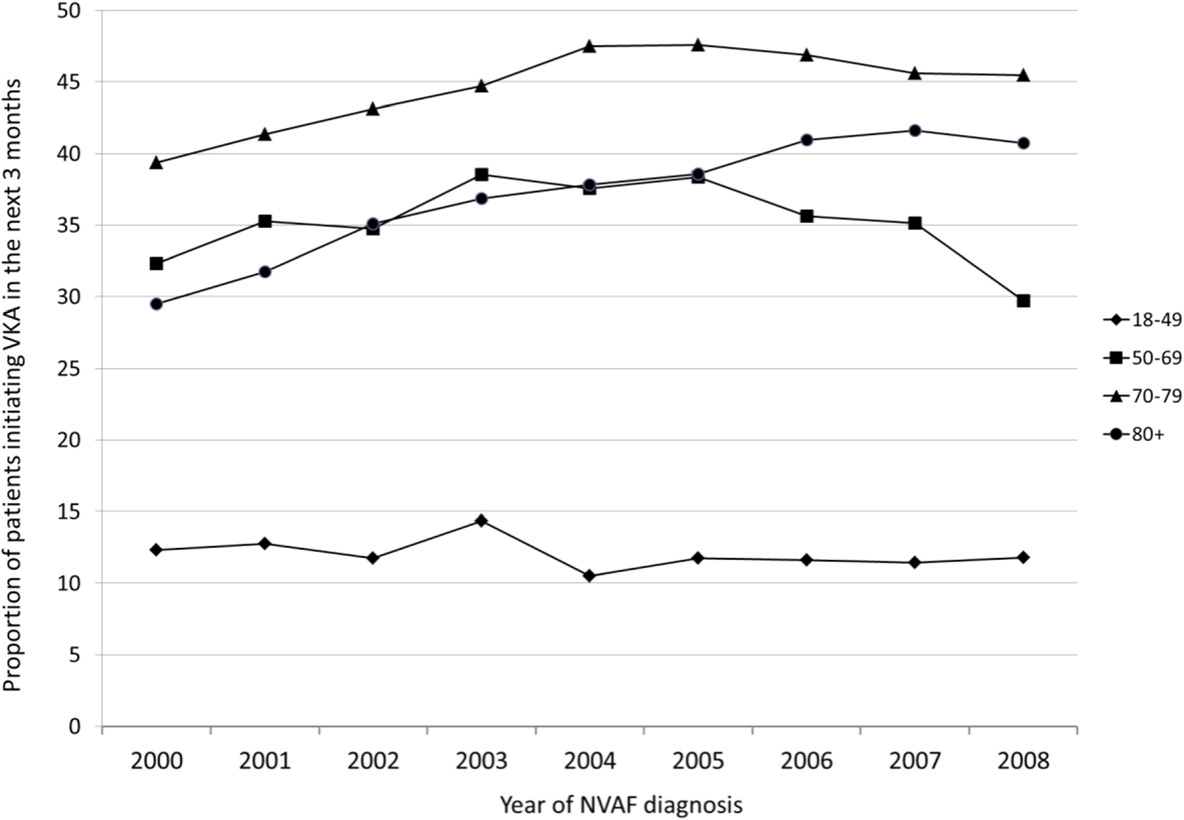
Proportion of patients initiating VKA within 3 months following NVAF diagnosis, stratified by age and calendar year of diagnosis

**Fig. 3:**
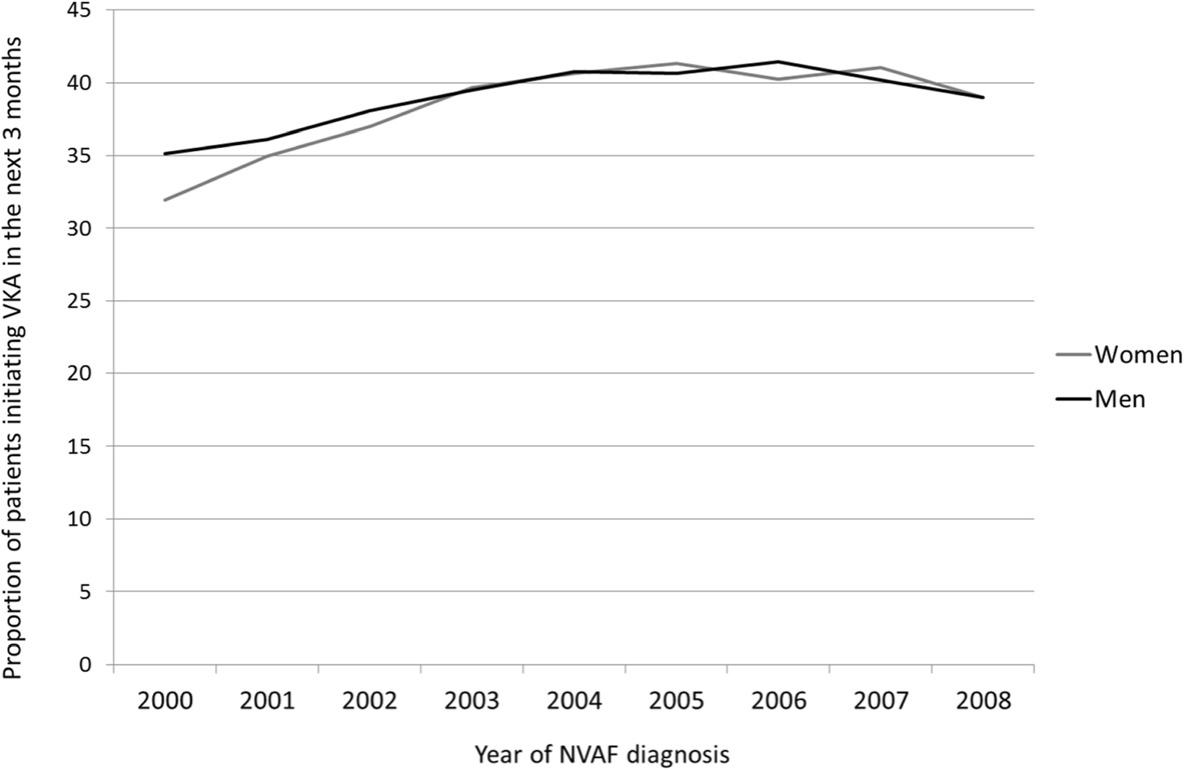
Proportion of patients initiating VKA within 3 months following NVAF diagnosis, stratified by sex

**Fig. 4:**
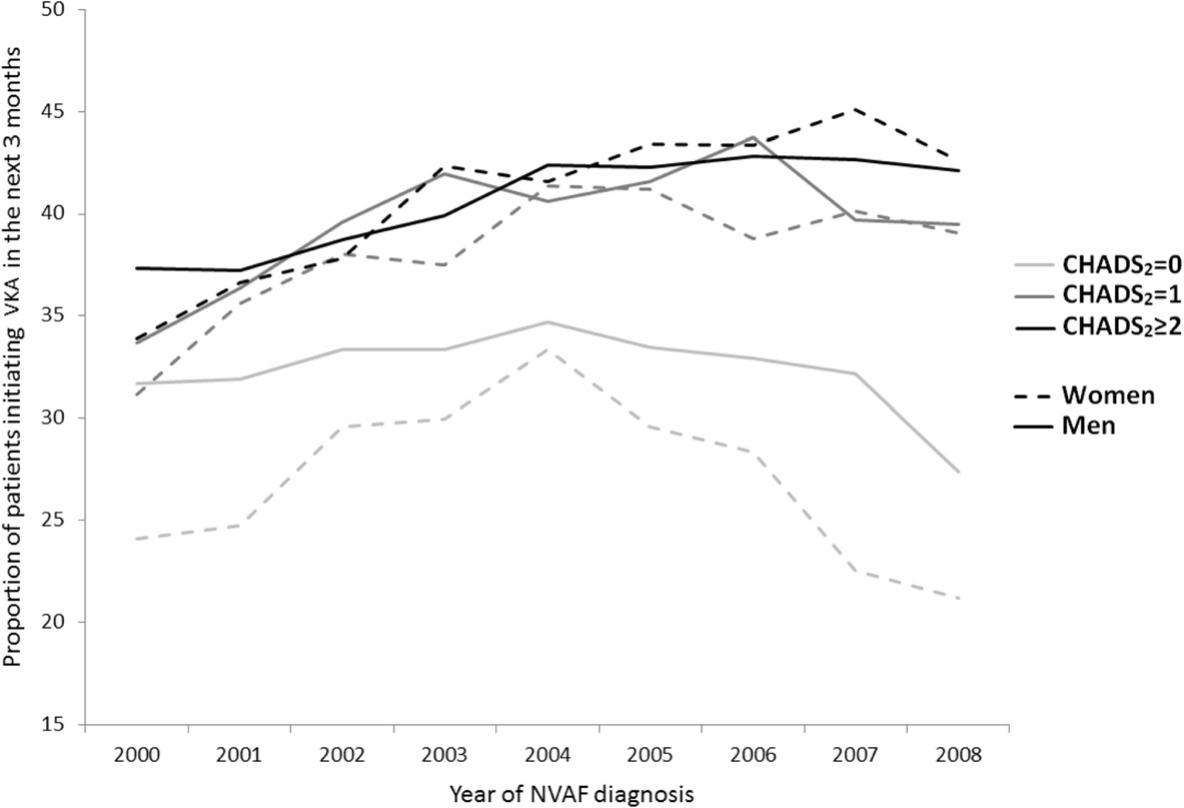
Proportion of patients initiating VKA within 3 months following NVAF diagnosis, stratified by sex, CHADS_2_ score, and calendar year of diagnosis

Following NVAF diagnosis, 47.1 % of the patients were prescribed VKA (5.9 % in combination with an antiplatelet and 15.3 % switching from antiplatelet to VKA or adding VKA), 35.5 % received an antiplatelet only, and 17.4 % did not initiate an antithrombotic therapy. The proportion of patients prescribed VKA increased with CHADS_2_ score. It was 37.8 % for a score of 0, 47.7 % for score of 1, and 49.1 % for patients with a CHADS_2_ score ≥ 2. Most patients initiating VKA did so in the 15 days following NVAF diagnosis, regardless of age, sex, and CHADS_2_ score. The proportion of patients initiating VKA within 3 months of diagnosis increased from 33.5 % to 39 % over the 10-year study period independently of age and CHADS_2_ score (p < 0.0001). This increased use of VKA was mainly driven by a higher proportion of treated patients aged 80 or more (from 29 % to 41 %) (Fig. 2). At the end of the study period, women were prescribed VKA as frequently as men shortly after NVAF diagnosis, except in the subgroup of patients with a low risk of ischemic stroke (Figs. 3 and 4). The hazard ratios of initiating VKA within 3 months of NVAF diagnosis according to baseline characteristics are presented in Table 2. Older patients as well as those with a previous ischemic stroke, congestive heart failure, or cardiovascular medication use were more likely to initiate VKA in the first 3 months after diagnosis. Men were also slightly more likely to initiate VKA shortly after NVAF diagnosis than women. However, when restricting the analysis to patients 75 years or older, there was no difference between men and women (HR 1.01, 95 % CI 0.99-1.04). As expected, patients with a prior bleeding event, dementia, liver disease, or chronic renal failure were less likely to initiate VKA.

**Table 2:** Hazard ratios of characteristics associated with VKA initiation within 3 months following NVAF diagnosis

	%	Crude HR	Adjusted HR (95 % CI)
Size, n	135,241		
Age			
18-29	0.45	0.22	0.23 (0.15 - 0.36)
30-39	0.83	0.45	0.45 (0.36 - 0.58)
40-49	2.06	1.00	1.00 (Reference)
50-59	5.01	1.73	1.68 (1.51 - 1.87)
60-69	16.81	2.73	2.55 (2.31 - 2.82)
70-74	17.43	3.29	3.05 (2.77 - 3.36)
75-79	20.29	3.51	3.28 (2.97 - 3.61)
80-84	18.06	3.31	3.12 (2.83 - 3.44)
85-89	12.09	2.76	2.66 (2.41 - 2.94)
≥90	6.97	1.60	1.57 (1.41 - 1.74)
Male	47.90	1.02	1.09 (1.07 - 1.11)
Comorbidities			
Hypertension	61.66	1.21	1.03 (1.01 - 1.05)
Myocardial infarction	20.64	0.94	0.89 (0.87 - 0.92)
Angina pectoris	20.02	0.94	0.88 (0.86 - 0.90)
Congestive heart failure	20.46	1.15	1.20 (1.18 - 1.23)
Prior stroke or TIA	8.62	1.22	1.25 (1.21 - 1.28)
Prior bleeding events			
Intracranial bleeding	1.00	0.56	0.55 (0.49 - 0.61)
Gastrointestinal bleeding	5.75	0.65	0.67 (0.64 - 0.70)
Other bleeding	8.84	0.85	0.85 (0.83 - 0.88)
Diabetes	25.39	1.08	0.97 (0.95 - 0.99)
Hyperlipidemia	42.47	1.18	1.00 (0.95 - 1.05)
Vascular disease	12.12	0.94	0.87 (0.84 - 0.89)
Chronic renal failure	10.70	0.89	0.87 (0.84 - 0.90)
Complications of alcohol abuse	3.61	0.61	0.78 (0.73 - 0.83)
Liver disease	2.53	0.67	0.80 (0.75 - 0.85)
Cancer	19.58	0.79	0.76 (0.75 - 0.78)
Coagulation defects	1.81	0.75	0.88 (0.82 - 0.95)
Predisposition to falls	15.44	0.75	0.80 (0.78 - 0.83)
Dementia/schizophrenia	8.81	0.55	0.65 (0.63 - 0.68)
Venous thromboembolism	5.47	1.37	1.48 (1.43 - 1.53)
COPD	26.65	0.94	0.93 (0.91 - 0.95)
Medications			
ACE	35.62	1.21	1.12 (1.10 - 1.15)
ARB	21.28	1.25	1.12 (1.09 - 1.14)
CCB	41.51	1.27	1.19 (1.16 - 1.21)
Diuretics	50.08	1.25	1.09 (1.07 - 1.12)
Antidepressants	19.44	0.84	0.91 (0.89 - 0.94)
Antipsychotics	5.81	0.61	0.83 (0.79 - 0.87)
NSAIDs	36.35	1.01	1.00 (0.98 - 1.02)
Statins	38.55	1.19	1.05 (1.00 - 1.10)

*Abbreviations*: *HR* hazard ratio, *TIA* transient ischemic attack, *COPD* chronic obstructive pulmonary disease, *ACE* angiotensin-converting enzyme inhibitors, *ARB* angiotensin receptor blockers, *NSAIDs* non-steroidal anti-inflammatory drugs

**Fig. 5:**
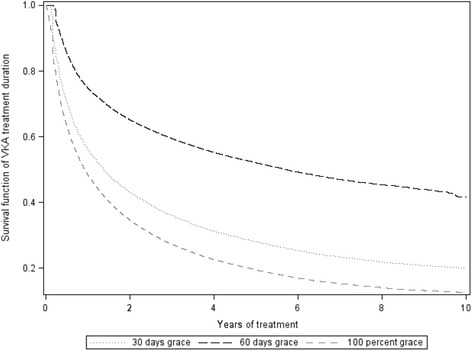
Survival estimates of the time from VKA initiation to first discontinuation according to the definition of the grace period

The estimated median time from VKA treatment initiation to the first discontinuation was 11.1 months (95 % CI 10.9- 11.4) for VKA (Fig. 5). However, 79 % of the patients who discontinued VKA had a new prescription after a median time of 35 days. In sensitivity analyses, changing the grace period to 30 and 60 days led to substantial variation in duration of VKA use until first discontinuation, with estimated medians of 1.4 and 5.7 years, respectively (Fig. 5). With a grace period of 60 days, still 61 % of the patients resumed VKA treatment after a median time of 87 days.

The median duration of VKA use until first discontinuation varied only slightly with CHADS_2_ score (data not shown). In multivariate analysis, older patients and those with a prior ischemic stroke or transient ischemic attack were less likely to discontinue VKA (Table 3). Restricting the analysis to patients 75 years or older, age and sex were not determinants of the first VKA discontinuation.

**Table 3:** Hazard ratios of determinants associated with VKA discontinuation in NVAF

Factors	%	Crude HR	Adjusted HR (95 % CI)
Size, n	63,675		
Age			
18-29	0.07	1.60	1.65 (1.19 - 2.29)
30-39	0.19	1.47	1.48 (1.19 - 1.84)
40-49	0.89	1.00	1.00 (Reference)
50-59	3.59	0.88	0.88 (0.79 - 0.98)
60-69	16.79	0.83	0.83 (0.76 - 0.92)
70-74	19.82	0.82	0.82 (0.75 - 0.91)
75-79	23.78	0.78	0.79 (0.72 - 0.87)
80-84	19.80	0.74	0.75 (0.68 - 0.83)
85-89	11.10	0.77	0.79 (0.71 - 0.87)
≥90	3.97	0.77	0.80 (0.71 - 0.88)
Time to treatment initiation			
0 day	39.51	1.00	1.00 (Reference)
1-7 days	23.73	1.07	1.11 (1.08 - 1.13)
8-30 days	12.11	1.00	1.04 (1.01 - 1.07)
31-90 days	5.99	1.02	1.04 (0.99 - 1.08)
>90 days	18.66	1.14	1.13 (1.10 - 1.17)
Male	48.46	1.08	1.03 (1.01 - 1.05)
Comorbidities			
Hypertension	64.50	0.98	0.98 (0.96 - 1.01)
Myocardial infarction	19.73	1.13	1.08 (1.05 - 1.11)
Angina pectoris	19.79	1.10	1.06 (1.04 - 1.09)
Congestive heart failure	21.15	1.06	1.02 (0.99 - 1.05)
Prior stroke or TIA	9.36	0.90	0.90 (0.87 - 0.93)
Prior bleeding events			
Intracranial bleeding	0.64	1.05	1.04 (0.92 - 1.18)
Gastrointestinal bleeding	4.44	1.13	1.06 (1.01 - 1.11)
Other bleeding	7.83	1.04	1.01 (0.97 - 1.04)
Diabetes	26.00	1.03	1.01 (0.99 - 1.04)
Hyperlipidemia	44.96	1.03	1.08 (1.03 - 1.13)
Vascular disease	11.52	1.10	1.06 (1.02 - 1.09)
Chronic renal failure	9.40	1.19	1.14 (1.10 - 1.18)
Complications of alcohol abuse	2.38	1.19	1.10 (1.03 - 1.17)
Liver disease	1.85	1.20	1.09 (1.02 - 1.17)
Cancer	16.72	1.14	1.13 (1.10 - 1.16)
Coagulation defects	1.42	1.16	1.08 (0.99 - 1.17)
Predisposition to falls	12.46	1.03	1.01 (0.98 - 1.04)
Dementia/schizophrenia	4.97	1.04	1.04 (0.99 - 1.09)
Venous thromboembolism	6.36	1.20	1.16 (1.12 - 1.21)
COPD	25.52	1.07	1.03 (1.01 - 1.06)
Medications			
ACE	38.49	0.99	0.97 (0.95 - 1.00)
ARB	23.56	0.99	1.00 (0.97 - 1.02)
CCB	45.28	0.99	0.98 (0.96 - 1.01)
Diuretics	53.37	0.99	1.00 (0.98 - 1.02)
Antidepressants	17.18	1.03	1.02 (0.99 - 1.04)
Antipsychotics	3.64	1.07	1.03 (0.97 - 1.08)
NSAIDs	37.56	1.02	1.01 (0.99 - 1.03)
Statins	40.93	1.02	0.92 (0.88 - 0.97)

*Abbreviations*: *HR* hazard ratio, *TIA* transient ischemic attack, *COPD* chronic obstructive pulmonary disease, *ACE* angiotensin-converting enzyme inhibitors, *ARB* angiotensin receptor blockers, *NSAIDs* non-steroidal anti-inflammatory drugs

A total of 4,621 patients (3.4 % of the patients in the cohort) experienced a major bleeding event in the year following NVAF diagnosis. Among them, 1,705 patients (36.9 %) had received a VKA prescription within one month preceding this bleeding event and 1,427 (30.9 %) an antiplatelet. Among those on VKA, 362 (21.2 %) died within 3 months following the major bleeding, 819 (48.0 %) received at least one VKA prescription, 279 (16.4 %) an antiplatelets prescription, and the remaining 245 (14.4 %) did not restart any antithrombotic treatment.

## Discussion

In this large population-based cohort of patients diagnosed with incident NVAF, around 40 % of patients initiated VKA in the year following diagnosis, mostly within 15 days after diagnosis. The proportion of patients initiating VKA shortly after diagnosis, i.e. in the first 3 months increased over the 10-year study period, with up to 39 % of patients diagnosed in 2008 being treated with VKA. This increased prescribing over time was greater for women and older patients. The main determinants of VKA initiation shortly after NVAF diagnosis were age and a history of cerebrovascular event. Overall, there were only minor differences in rates of initiation or discontinuation of VKA between men and women, especially in older patients.

Underuse of oral anticoagulation therapy in patients with atrial fibrillation has been constantly reported in the literature with recent estimations of patients treated with VKA ranging from around 40 to 60 % in large cohorts [9–17]. Even in patients considered at high risk for ischemic stroke, such as patients with a CHADS_2_ score of 2 or more, less than 70 % of patients were treated with VKA according to a recent systematic review when most of these high-risk patients should be treated with anticoagulants based on all current guidelines [18]. Our results based on a recent large cohort of patients with incident NVAF are in line with these previous estimates. Indeed, only 49 % of patients with a CHADS_2_ score of 2 or more were prescribed VKA in our cohort. Moreover, given that around 60 % of patients in our cohort were at high risk of stroke (CHADS_2_ of 2 or more) and 28 % were at intermediate risk, at least 60 % of our cohort overall would be expected to be treated with oral anticoagulants using a conservative estimate. We were able to examine prescribing trends over time, and we found an increased use of anticoagulants after NVAF diagnosis over the last decade, which was not explained by the aging of the population or a different vascular risk profile in patients diagnosed more recently. This increased proportion of treated patients was mainly driven by an increase use in older patients, those most likely to benefit from oral anticoagulation. A recent British cohort study of 81,381 patients aged 60 years or older also showed a moderate increased prescribing of VKA in patients with AF over the same 10-year study period [16]. Similarly to our finding, the highest increased was seen in patients over 70 years old at diagnosis. It is therefore possible that studies repeatedly showing the benefit of anticoagulation among older patients with NVAF, and increased awareness of physicians through published guidelines have led to improvement in the management of NVAF in this population. However, anticoagulants remained underused and the recent introduction of new oral anticoagulants on the market may further increase the proportion of patients with NVAF prescribed anticoagulation in light of their ease of use.

Sex differences in patterns of VKA use have been reported in several previous studies. Indeed, it has been showed that women, although at higher risk of ischemic stroke in the context of AF, were less likely to be treated with oral anticoagulant following NVAF diagnosis than men [16, 17, 19]. However, in our study, women were only slightly less likely to be prescribed VKA compared to men, in accordance with a recent systematic review and meta-analysis reporting a pooled odds ratio of 1.12 for the association between male sex and warfarin use [20]. More importantly, we found that the proportion of women initiating VKA after NVAF diagnosis increased over the decade, particularly in patients with a moderate to high risk of ischemic stroke so that there was no more sex difference at the end of the study period in these subgroups. A recent Canadian study restricted to patients aged 65 or older and hospitalised with an AF diagnosis in Québec between 1998 and 2007 found very similar warfarin initiation between men and women in a cohort of 83,513 patients [9]. However, the study examined prescriptions issued in the 30 days post discharge only and did not examine trends over time.

Estimation of discontinuation of VKA in large cohorts of patients initiating anticoagulation after NVAF diagnosis varies substantially in the literature. For instance, the estimated median time to the first discontinuation of VKA was 127 days in a large US cohort of 116,969 patients with NVAF. [17] In a British cohort of 41,910 patients with chronic AF, 30 % had discontinued warfarin within one year after treatment initiation [21]. Among 4188 patients newly starting warfarin in the anticoagulation and risk factors in Atrial Fibrillation Study, 26.3 % discontinued warfarin within the first year of therapy [22]. These differences are likely explained by different definitions of VKA interruption. Indeed, we showed that the time to first treatment discontinuation varied greatly according to the grace period definition with an estimated median time of several years when using a grace period of 60 days as opposed to a median time of 11 months when using a 100 % grace period which corresponds to less than 15 days for most patients in our cohort. This variation suggests that VKA discontinuation is often temporary rather than definitive, and that many patients continue oral anticoagulation treatment once initiated. Reasons for temporary interruptions of anticoagulation are numerous and include in particular temporary interruption for minor bleeding or planned procedure or surgery. Moreover, time to VKA treatment discontinuation may be artificially shortened when measured using administrative databases since duration of each prescription is imprecise and VKA treatment is frequently prone to doses adjustments. In the ATRIA study, around 25 % of patients had evidence of restarting warfarin within 2 years after the first discontinuation, and overall the proportion of patients prescribed warfarin remained relatively unchanged after the first year [22]. In our study, using a stricter definition of discontinuation, the percentage of patients resuming VKA was even higher. Nonetheless, a non-negligible number of patients discontinue VKA and even temporary interruptions are of concern as they are associated with an increased risk of thromboembolic event. In this context, new oral anticoagulants may help improve the long-term compliance to anticoagulation therapy.

Our study is based on one of the largest and most recent population-based cohort of patients diagnosed with NVAF, allowing precise and contemporary estimates of VKA treatment patterns in a real-world setting. Moreover, our study follow-up covered the entire last decade so that we were able to examine recent time trends in antithrombotic treatment just preceding the introduction of new oral anticoagulants. Data for each patient, including prescriptions, were collected prospectively, thus avoiding the potential for recall bias inherent in field studies. Moreover, the use of drugs was not simply based on written prescriptions, but rather on dispensed prescriptions actually filled by patients, which reduces significantly misclassification of exposure. All patients in our study had drug coverage from the province of Québec; therefore we likely captured most antithrombotic prescriptions. However, we cannot exclude the possibility that some prescriptions were issued over the counter especially for aspirin or out of the provincial system for VKA, but this number is likely to be small in the context of a chronic disease in patients with full provincial drug coverage. Also, our study describes the out-patients prescriptions patterns as we did not have access to in-patients prescriptions. This may potentially leads to underestimate the time to the first antithrombotic treatment discontinuation if some patients have their treatment renewed while in hospital. However, this is unlikely to change our results as the number and length of hospitalisations were quite small in the year following NVAF diagnosis in our cohort study. Finally, using an administrative database such as RAMQ, the diagnosis of AF was based on ICD codes only and we were not able to validate the NVAF diagnosis with electrocardiogram or other medical data. Also, ICD 9 and 10 codes for AF do not differentiate between paroxysmal and chronic AF. However, using the same cohort, we previously found incidence rates of NVAF very similar to other contemporary cohorts using various diagnostic criteria [23], suggesting that our cohort likely includes mostly true incident cases of NVAF. Similarly, the identification of patients’ comorbidities were based on ICD codes without access to medical records and some clinical data such as smoking and obesity are not available in the RAMQ database.

## Conclusions

In conclusion, findings from this large population-base cohort of patients with NVAF indicate that anticoagulation is still underused even though the efficacy of VKA in preventing thromboembolic events has been well established. However, there was an increased proportion of patients initiating VKA over the last decade, particularly for women and older patients. Further research will clarify whether the introduction of new oral anticoagulants improve the overall therapeutic management of patients with NVAF.

## Ethics approval

The study protocol was approved by the Research Ethics Committee of the Jewish General Hospital, Montreal, Canada (Number 11-076).

## Consent for publication

Not applicable.

## Availability of data and materials

No additional data available.

## Abbreviations

NVAF: non-valvular atrial fibrillation
RAMQ: Régie de l'assurance maladie du Québec
VKA: vitamin K antagonists
